# Differences in prey personality mediate trophic cascades

**DOI:** 10.1002/ece3.6648

**Published:** 2020-08-12

**Authors:** Nathalie R. Sommer, Oswald J. Schmitz

**Affiliations:** ^1^ School of the Environment Yale University New Haven CT USA

**Keywords:** consistent individual differences, food webs, habitat domains, intraspecific variation, predator–prey interactions

## Abstract

Functional trait approaches in ecology chiefly assume the mean trait value of a population adequately predicts the outcome of species interactions. Yet this assumption ignores substantial trait variation among individuals within a population, which can have a profound effect on community structure and function. We explored individual trait variation through the lens of animal personality to test whether among‐individual variation in prey behavior mediates trophic interactions. We quantified the structure of personalities within a population of generalist grasshoppers and examined, through a number of field and laboratory‐based experiments, how personality types could impact tri‐trophic interactions in a food chain. Unlike other studies of this nature, we used spatial habitat domains to evaluate how personality types mechanistically map to behaviors relevant in predator–prey dynamics and found shy and bold individuals differed in both their habitat use and foraging strategy under predation risk by a sit‐and‐wait spider predator. In the field‐based mesocosm portion of our study, we found experimental populations of personality types differed in their trophic impact, demonstrating that prey personality can mediate trophic cascades. We found no differences in respiration rates or body size between personality types used in the mesocosm experiment, indicating relative differences in trophic impact were not due to variation in prey physiology but rather variation in behavioral strategies. Our work demonstrates how embracing the complexity of individual trait variation can offer mechanistically richer understanding of the processes underlying trophic interactions.

## Graphical abstract

We explored individual trait variation through the lens of animal personality to test whether among‐individual variation in prey behavior mediates trophic interactions. Unlike other studies of this nature, we used spatial habitat domains to evaluate how shy and bold personality types mechanistically map to behaviors relevant in predator‐prey dynamics. We found personality types differed in their habitat use and foraging strategy under predation risk and, by experimentally manipulating personality types within a tritrophic food web, we found differences in prey personality mediate trophic cascades.

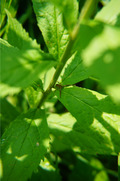

## 1 INTRODUCTION

The classic approach to studying trophic dynamics at the level of species interactions is increasingly giving way to examinations of species' functional traits (McGill, Enquist, Weiher, & Westoby, 2006). This shift in focus has come about because of the recognition that the degree of trait variation between interacting species can explain considerable variation in the nature and strength of trophic interactions (McGill et al., 2006; Post, Palkovacs, Schielke, & Dodson, 2008; Schmitz, Buchkowski, Burghardt, & Donihue, 2015). Yet, functional trait approaches have largely proceeded by assuming that mean trait value sufficiently characterizes species interactions (Schmitz et al., 2015). This assumption may not hold if differences in functional traits among individuals within a population have a decided effect on the nature and strength of trophic structure and function (Figure 2; Benesh & Kalbe, 2016; Bolnick et al., 2011; Hazard, Kruitbos, Davidson, Taylor, & Johnson, 2017; Lichtenstein, Chism, Kamath, & Pruitt, 2017; Okuyama, 2008; Ovadia & Schmitz, 2002; Post et al., 2008; Schmitz et al., 2015; Start & Gilbert, 2017).

**FIGURE 1:**
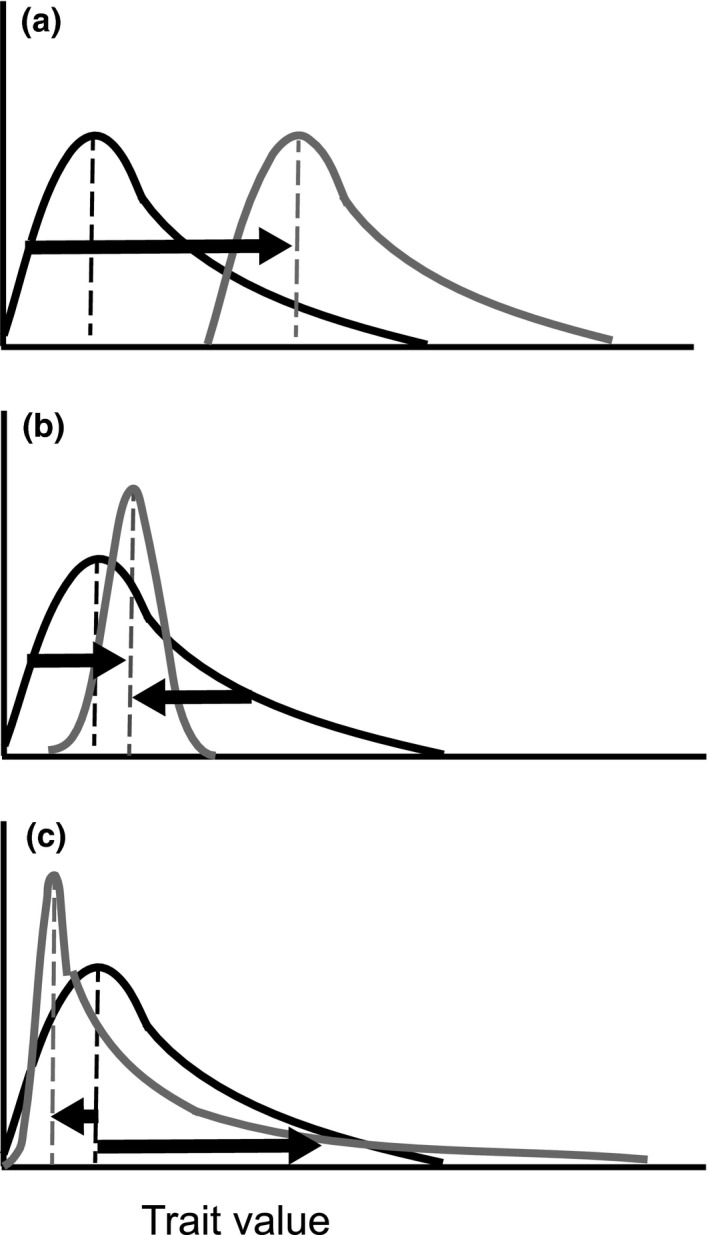
A schematic demonstrating how trait mean may not appropriately characterize species interactions. Frequency distributions represent individual variation for any given trait within a population. The black shows an initial trait distribution, and the light gray signifies a change in the trait distribution corresponding to a change in environmental context. (Panel a) If all individuals have an identical trait response, the mean is an accurate characterization of the population's heterospecific interactions. (Panel b) If individuals do not have an identical trait response but converge toward a similar mean, the resulting trait distribution has less variance and a shifted mean value within the population. (Panel c) If individuals do not have an identical trait response and instead have opposing directional responses, the resulting distribution has more variance and a shifted mean within a population

The mean (expected) net effect of a species will reflect the mean (expected) value of the functional trait only if all individuals in the population maintain the same direction and magnitude of trait response to any given environmental context (Figure 1a). The mean value will not capture differences among individuals if trait responses are not identical (e.g., dependent on physiological or behavioral states; Bolnick et al., 2011; Ovadia & Schmitz, 2002; Pettorelli, Hilborn, Duncan, & Durant, 2015; Schmitz & Trussell, 2016), potentially to the extreme degree that responses may occur in opposing directions (Figure 1c) which would shift the mean and increase functional trait variance. Alternatively, nonidentical responses to the same environmental context may converge to a similar trait value, decreasing within population variance (Figure 1b). The potential for variable responses among individuals implies that functional trait variation within populations is just as important as functional trait variation among species within a community (Des Roches et al., 2018; Rall, Kalinkat, Ott, Vucic‐Pestic, & Brose, 2011; Rudolf & Rasmussen, 2013). Programmatic averaging in ecology has also been criticized due to Jensen's inequality (Denny, 2017; Ruel & Ayres, 1999; Welsh, Peterson, & Altmann, 1988), which states if a relationship is nonlinear, the average of the function will not be equal to the function of the average (Jensen, 1906). Unless a relationship is linear, it is inadequate to use the average to make general predictions about any one ecological outcome (Bolnick et al., 2011; Denny, 2017; Okuyama, 2008). Functional trait variation has consequences for evolutionary processes underpinning trophic interactions (e.g., Cortez, 2016). If ecology and evolution indeed occur on commensurate timescales (Hairston, Ellner, Geber, Yoshida, & Fox, 2005), ignoring functional trait variation within populations stymies our understanding of how species may respond to rapid environmental change. For these reasons, understanding how variation in functional traits drives both intra‐ and interspecific interactions will improve our ability to mechanistically scale from populations to ecosystem processes (Belgrad & Griffen, 2016; Bolnick et al., 2011; Schmitz, 2017; Schmitz et al., 2015; Sih, Cote, Evans, Fogarty, & Pruitt, 2012; Start & Gilbert, 2017).

Within a population, individuals can differ in fundamental functional traits—their behavioral responses to different environmental contexts. Behavior is usually considered a highly labile trait for all individuals, but empirical work within the last decade has demonstrated behaviors of individuals can be constrained (Chang, Teo, Norma‐Rashid, & Li, 2017; Eccard & Herde, 2013; Gyuris, Feró, & Barta, 2012; Parthasarathy, Joshi, Kalyadan, & Somanathan, 2019). Correlated, constrained behaviors that are consistent within an individual across contexts have been termed a “personality” trait (Biro & Stamps, 2008; Dingemanse, Kazem, Réale, & Wright, 2010). Individual differences in personality are known to alter both direct (Belgrad & Griffen, 2016; Toscano & Griffen, 2014) and indirect (Griffen, Toscano, & Gatto, 2012) effects across trophic interactions (reviewed in Toscano, Gownaris, Heerhartz, & Monaco, 2016). For example, predator personality can impact the direction and strength of trophic cascades by determining patterns of attack on herbivore prey (Start & Gilbert, 2017). While trophic consequences of predator behavioral variation are becoming increasingly well understood (Start & Gilbert, 2017; Toscano & Griffen, 2014), concomitant understanding of how prey personality mediates trophic interactions lags considerably (but see Griffen et al., 2012; Toscano, Lichtenstein, & Costa‐Pereira, 2020), thereby giving incomplete understanding of the role of individual behavioral variation in trophic interactions (Belgrad & Griffen, 2016).

We report on a study that evaluated how personality differences in a species of herbivore prey mediated tri‐trophic interactions involving its predator and its plant resources. We used a combination of laboratory and field experiments to develop a comprehensive understanding of how differences in prey personality scale to influence the nature and strength of trophic interactions. Our study was designed bearing in mind common critiques of personality research, namely whether personality is effectively any different from animal physiology (Beekman & Jordan, 2017) and whether there is any relevant ecological interpretation of any one personality type (Carter, Feeney, Marshall, Cowlishaw, & Heinsohn, 2013; Moirón, Laskowski, & Niemelä, 2020). Unlike other studies of this nature (Lichtenstein et al., 2017; Start & Gilbert, 2017), we take a deeper mechanistic approach by evaluating how personality types map directly to ecologically pertinent behaviors using a spatial habitat domain approach. Spatial habitat domains are detailed time budgets that track an individual's foraging activity, microhabitat use, and spatial extent of movement (Guiliano, Karr, Sommer, & Buchkowski, 2020; Miller, Ament, & Schmitz, 2014; Northfield, Barton, & Schmitz, 2017; Rosenblatt, Wyatt, & Schmitz, 2019). Guided by the basic theory of occupancy models for larger, wider ranging species, our spatial habitat domain approach elucidates both the full spatial extent of movement and the core areas of habitat use for individuals within the vertical grassland canopy.

## 2 METHODS

### 2.1 Overview

We conducted a series of laboratory and field experiments between June and September 2018. Our first goal was to construct a trait distribution for personality within a population of grasshopper herbivores. We assayed over 500 individual grasshoppers for personality, and from the distribution, we designated the lower quartile of individuals as “shy” and the upper quartile as “bold”. We then took a subset of those shy and bold individuals (*n* = 40) for detailed assessments of physiology (respiration rates) and spatial habitat domains. Respiration rates were aimed to disentangle physiology from personality (Careau, Thomas, Humphries, & Réale, 2008), and habitat domain observations were aimed to contextualize personality within ecologically pertinent behaviors. Using the remaining individuals from the distribution in the respective shy and bold quartiles (*n* = 200), we created experimental populations for a field mesocosm experiment to assess how personality might mediate trophic cascades. Finally, we collected additional individuals, not within the assayed distribution, to assess lifetime consistency of personality in the laboratory.

### 2.2 Study site

**FIGURE 2:**
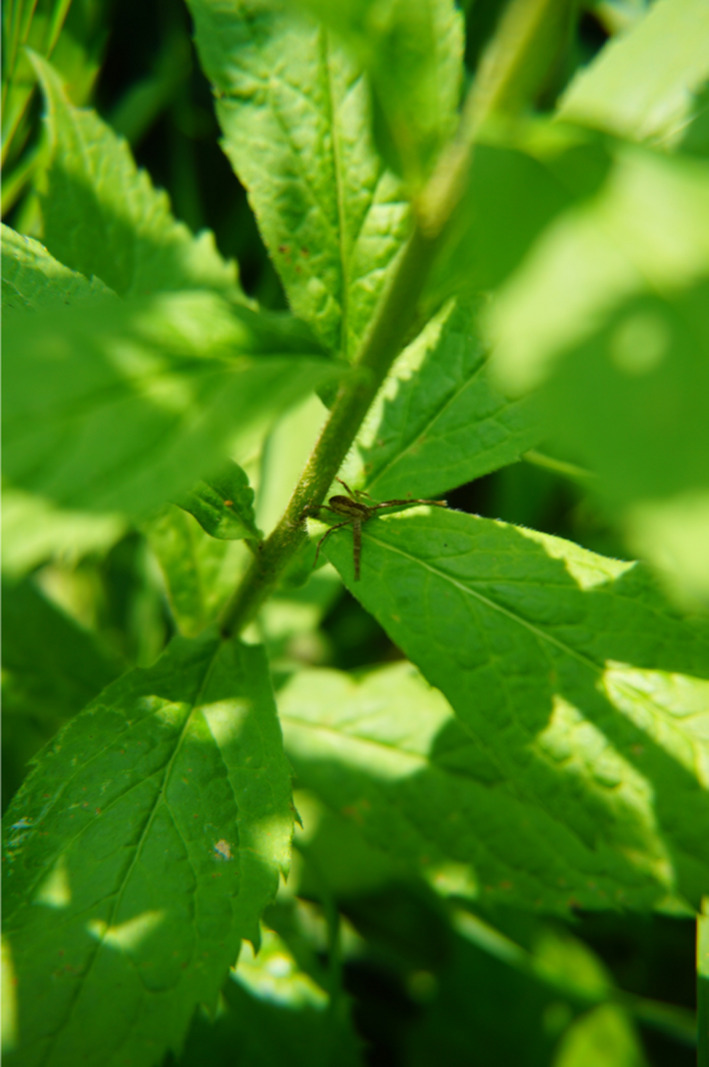
*Pisaurina mira* perched on *Solidago rugsa* at Yale‐Myers Forest

The field research was performed in old‐fields around Yale‐Myers Research Forest in northeastern Connecticut, USA. Old‐fields are legacies of abandoned colonial agriculture that have been maintained in open conditions (Foster, 1992). The important species in old‐fields are effectively represented in three trophic levels: (a) sit‐and‐wait spider predator *Pisaurina mira* (Fam. Pisauridae), (b) dominant generalist herbivore grasshopper *Melanoplus femurrubrum* (Fam. Acrididae) and (c) plant functional groups *Solidago*, grasses, and forbs (Figure 2). Previous research has demonstrated that *P. mira* spider predators have predominantly nonconsumptive effects on their *M. femurrubrum* grasshopper prey (Beckerman, Uriarte, & Schmitz, 1997; Schmitz, Beckerman, & O'Brien, 1997). Grasshoppers mediate the top‐down effects of these spider predators via changes in foraging and habitat selection (shifting from eating relatively protein‐rich grasses to eating and seeking refuge in relatively carbohydrate rich *Solidago*), and changes in physiological responses to perceived predation risk (increasing grasshopper demands for carbohydrates and enhancing predation‐escape performance; Hawlena & Schmitz, 2010b; Hawlena et al., 2011; Schmitz & Suttle, 2001). The three main functional groups of plants are (a) grasses *Poa* spp. and *Phleum pratense* which provide grasshoppers a high source of protein, (b) *Solidago rugosa* Mill. (goldenrod) which provides refuge and a source of carbohydrate energy, and (c) a variety of other old‐field herb species whose abundances are modified by grasshopper herbivory on the competitive dominant goldenrod (Schmitz, Buchkowski, Smith, Telthorst, & Rosenblatt, 2017).

We collected all *M. femurrubrum* individuals from a single field that had no spider predators of this grasshopper species (*Phiddipus* spp., *Gladicosa gulosa*, *Pisaurina mira*, or *Rabidosa rabida*), confirmed by systematically surveying the field prior to collection and twice more over the duration of the field experiment. This was done in an effort to minimize experiential bias from predator encounters during early instars. We caught third‐instar grasshoppers in early July using sweep nets and immediately transported them to the laboratory at Yale Myers Research Forest. All individuals (*n* = 510) were weighed and then housed individually in 24 × 16 × 16 cm plastic terraria. Individuals were provided ad libitum supply of freshly clipped grass and *Solidago*. We misted vegetation with water every 24 hr to simulate typical patterns of dew. The laboratory at Yale Myers Research Forest is not climate controlled and has floor‐to‐ceiling windows; laboratory conditions reflected ambient field temperatures and light conditions.

### 2.3 Intraspecific variation in personality

Personality is often considered along a shy‐to‐bold continuum (Reale, Reader, Sol, McDougall, & Dingemanse, 2007; Wilson, Clark, Coleman, & Dearstyne, 1994), quantified using different behavioral assays repeated on a single individual (Reale et al., 2007; Sih, Bell, & Johnson, 2004; Wolf & Weissing, 2012). The shy–bold continuum represents important functional trait variation mediating trophic interactions, because it reflects the degree of apprehension exhibited by an individual toward environmental stressors such as predation risk. Consequently, the degree of shyness or boldness may determine the degree to which individuals make the tradeoff between acquiring nutrients from foraging and avoiding predation risk (Wilson et al., 1994). Bold individuals ought to accept a high level of risk and forage to maximize nutrient (protein) gain, whereas shy individuals ought to avoid risk and thereby realize lower nutrient gain (Schmitz, 2017). Thus, we used shy–bold personality assays as a metric representing the relative tradeoff between foraging efforts and perceived predation risk (Reale et al., 2007).

With due consideration to the functional tradeoffs made by an herbivore in this food chain, we designed three standardized assays to measure the level of grasshopper apprehension related to the foraging‐risk tradeoff across widely different contexts: (a) foraging activity; (b) exploration; and (c) predation risk. While not mutually exclusive, the assays have different ecological interpretations (Reale et al., 2007). Foraging activity is a measure of nutrient acquisition and could be considered a baseline in which there is no risk and high reward. Exploration assays measure the degree of apprehension when encountering novel situations. Perceived predation risk is a true test of “boldness,” risking lethal consequences for a potential nutritional reward. We used these three assays to yield measures of personality along a functional continuum of shy to bold (Koolhaas et al., 1999; Wilson et al., 1994).

Prior to conducting the assays, we let individuals acclimate for 24 hr with food followed by a 12 hr fast. To measure activity level as Euclidean distance, we placed 1 cm squared grid under the 24 × 16 × 16 cm terrarium and set an assay‐specific object in the origin (or 0, 0) grid position. This standardized the location of the object while randomizing the starting grid position of the grasshoppers in the terrarium. For (a) foraging activity, the object was a dish of freshly cut vegetation; for (b) exploration, the object was a vertical plastic tube; for (c) perceived predation risk assay, the object was the same plastic tube plus a sit‐and‐wait spider predator *P. mira* held within it. Once the assay‐specific test objects were placed into the terraria, we allotted a 2 min acclimation period followed by a 15 min observation period. We recorded the location of the grasshopper, identified down to the individual 1 cm grid cell occupied by the grasshopper's head, every minute for the entire 15 min. At the end of each assay, we removed the test‐specific object and waited 15 min before beginning the next assay. Each 15 min assay was conducted only once on each individual and in the same order to minimize variation due to (i) state‐dependency caused by cycles of feeding and fasting, (ii) learning or carryover effects, (iii) exhausting the individual with multiple assays, or (iv) allowing it to take on a laboratory syndrome.

We ascertained if individuals were displaying a personality trait by determining whether or not activity level (as Euclidean distance) was repeatable for each individual within and across assay contexts (Assendorpf, 1990; Nakagawa & Schielzeth, 2010; Stoffel, Nakagawa, & Schielzeth, 2017). We partitioned each 15 min behavioral assay into three, 4 min periods. A 15‐min observation period yields 14 measures of movement; the first 2 min were not included in the partitioning to estimate repeatability, functionally extending the acclimation period to a total of 4 min. This approach to partitioning the observations does not give multiple, wholly independent measures of behavior; however, personality is most accurately represented with multiple observations of behavior within one context and across contexts (Dingemanse & Niemela, 2018). To estimate repeatability, we used generalized linear mixed models fit with 500 bootstrap iterations and a Poisson distribution in the rptR package (Stoffel et al., 2017). Given the limitation of this partitioning approach, repeatability estimates based on the full 15 min observation period are also reported in the Appendix S1.

**FIGURE 3:**
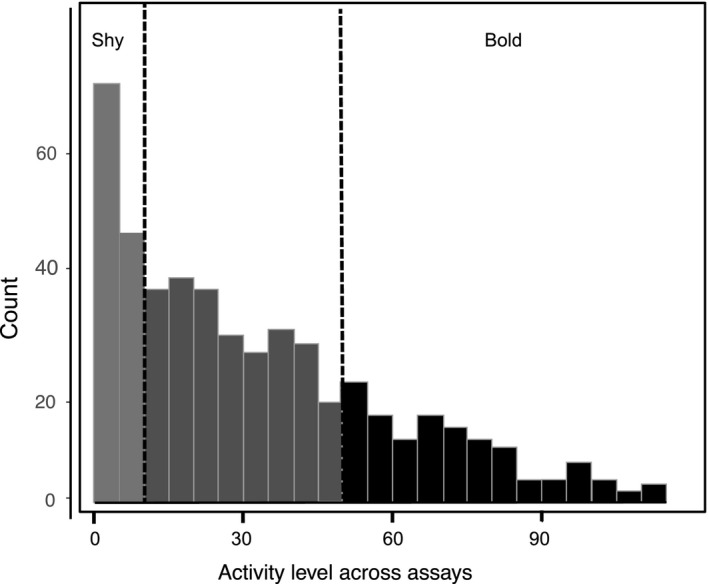
The distribution of average activity level for 510 individuals across the three behavioral assays was not normally distributed. We defined individuals in the lower quartile of the distribution as shy and individuals in the upper quartile as bold; hence, average activity level is described as the personality trait. As a consequence of this skewed distribution, there was more variation in personality trait value within the bold group than the shy group.

Acknowledging that shy–bold personality occurs along a continuum, we assigned a personality trait value to each individual by taking the mean activity level for each of the three, 15 min assays. We verified that mean activity level was not correlated with body size (*r* = −.082, *p* > .05). We then calculated a frequency distribution of personality trait values for all assayed individuals (Figure 3) and from that distribution, we defined “shy” individuals as those in the lower‐most quartile and “bold” individuals as those in the upper‐most quartile. Out of our sampled population of 510 individuals, we selected individuals from the respective quartiles for two experiments: (a) benchtop experimentation to evaluate shy/bold spatial movement in a simulated old‐field vegetation canopy and (b) field experimentation to evaluate the nature and strength of trophic cascades.

### 2.4 Spatial habitat domains

Habitat domain encompasses an individual's microhabitat and the spatial extent of movement within that habitat (Miller et al., 2014; Rosenblatt et al., 2019; Schmitz, 2005). Sit‐and‐wait *P. mira* predators tend to use a narrow habitat domain within the upper canopy, while *M. femurrubrum* prey tend to use a wider habitat domain slightly lower in the canopy (Barton & Schmitz, 2009; Schmitz & Suttle, 2001). Previous work has demonstrated that *M. femurrubrum* alter their habitat domain according to the hunting mode of the predator (Miller et al., 2014); by using a smaller area when *P. mira* are present, prey reduce the likelihood of fatal encounters. Examining habitat domain differences among individuals offers a mechanistic link to the consequences of intraspecific behavioral variation among prey in trophic interactions.

We selected 20 shy and 20 bold individuals from our distribution of 510 and examined their habitat domains under different predation contexts. Individuals were selected for similarity in body mass and trait score within their shy/bold classification. We placed each individual in a standard 40 × 30 × 88 cm benchtop microcosm constructed with a plywood base and insect screen stapled to wooden supports (Miller et al., 2014). Microcosms were filled with sod comprised of vegetation from the grasshoppers' native old‐field. We cut sod and adjusted vegetation as needed to equalize among all of the microcosms the amounts of the major plant functional groups, including three stalks of *Solidago* of equivalent height. The center of the microcosm contained a mesh tube (10 cm diameter × 88 cm height) with an additional stalk of *Solidago* to hold a spider predator, so as to produce predation risk cues but prevent grasshopper prey capture. We recorded the canopy height, substrate, and foraging behavior of grasshopper individuals every 30 min for 12 hr (0700–1900) when most foraging occurs for this predator and prey species (Miller et al., 2014). One day of observation served as the baseline (no‐predator control), with mesh tubes present in the microcosm, but empty. Following a rest day, a second day of observation assessed habitat domain under perceived predation risk, with each mesh tube containing one similarly sized sit‐and‐wait *P. mira* spider predator. Spiders were stocked into the mesh tubes and fasted for 24 hr before observation to ensure they went to typical hunting locations on the *Solidago* stalks.

We calculated spatial habitat domain for grasshoppers within our microcosms using established protocols (Northfield et al., 2017; Rosenblatt et al., 2019). We first binned the occurrence of individuals into 5 cm vertical height increments and then aggregated by personality type. Data for each bin were converted to relative frequency by normalizing count per bin by the total number of observations for all bins. We then calculated the habitat domain based on utilization distributions using a kernel density method to obtain the 50% isopleth (core utilization) and the 95% isopleth (broad area of utilization), which is the convention for spatial estimates of species habitat use (Fieberg & Kochanny, 2005). We evaluated the overlap of the utilization distributions of shy and bold individuals using the recommended Bhattacharyya's affinity (BA) formula (Carroll et al., 2019; Fieberg & Kochanny, 2005):

$$\text{BA}=\sum_{i=1}^{16}\sqrt{p_{i_{\text{shy}}}}\times \sqrt{p_{i_{\text{bold}}}}$$

where *i* is the vertical height bin and *p_ix_* is the relative frequency of use of the *i*th height bin by shy and bold individuals. Because there were no occurrences of grasshoppers in the lower portions of the microcosms, the total number of height bins was 16. While BA is a useful comparative metric, it is not a statistical test. Therefore, we also performed a chi‐square test of independence on areas of core utilization between the shy and bold groups using the counts for each vertical height bin.

### 2.5 Respiration rates

Once habitat domain observations were completed, we removed the spider predator from the microcosm and allotted a 48 hr rest period for the grasshoppers to reduce carryover effects. We then collected all grasshoppers from the microcosms and measured two respiration rates—a baseline and a predation risk scenario—to test for the potential that individual physiology, rather than individual personality, innately explains all differences among individuals (Beekman & Jordan, 2017; Careau et al., 2008). Single individuals were placed into a respiration measurement chamber, and the chamber was covered with a translucent cloth to reduce stress and disturbance. We allotted a 3 min acclimation period before a 5 min measurement period. After this baseline measurement, we placed a *P. mira* spider into an adjacent chamber and covered both chambers with the same translucent cloth such that the arthropods were exposed to reciprocal visual cues without other environmental interferences. We then allotted a second 3 min acclimation period followed by a 5 min measurement period. Visual cues are a minimal way to generate a perceived predation risk scenario because natural risk would include chemical signaling; however, given the limited nature of our closed‐system respirometer, visual cues were the closest estimable proxy. In addition, previous work with terrestrial arthropods has demonstrated that predator visual cues can induce oxidative stress (Janssens & Stoks, 2013). Respiration rate was calculated as the rate of carbon dioxide release from an individual over the 5 min measurement period, standardized by individual mass. We measured CO_2_ release using an incurrent flow‐through system (Q‐ S151 model, 1 ppm resolution; Qubit Biology Inc.) with an airflow rate of 200 ml/min for the CO_2_ analyzer. For analyses, we transformed the CO_2_ analyzer readings to adjust for ambient temperature and individual mass. Respiration rate was calculated as µl CO_2_/g/min (Hawlena & Schmitz, 2010b; Rosenblatt, Crowley, & Schmitz, 2016).

### 2.6 Lifetime consistency in personality

We collected and assayed an additional 30 individuals, not sourced from the originally sampled distribution of 510 individuals, to ascertain if there was consistency in personality measures across the entire lifetime of an individual. These 30 individuals were permanently housed in 24 × 16 × 16 cm individual plastic terraria at the Yale‐Myers Forest laboratory, which reflected ambient light and temperatures. We provided water ad libitum and changed vegetation within the terraria (freshly clipped grass and *Solidago*) every week or more frequently if vegetation appeared dried out or depleted. We assayed personality of individuals every 10 days following the same methods as detailed above, starting in early July and ending with the culmination of the field experiment (described below) in September. We did not classify individuals as shy and bold, rather we estimated behavioral repeatability within assay types, across assay types, and over time. We note here that the 15‐min assay for each context was also partitioned into three, 4‐min observations to estimate repeatability, and the repeatability estimate for the full 15‐min assay is similarly reported in the Appendix S1.

### 2.7 Personality and trophic impact

In late May, prior to collection of individual grasshoppers and personality assessments, we placed standard cylindrical field mesocosms (0.25 m^2^ × 1.0 m) over naturally growing vegetation in an old‐field at Yale‐Myers Forest. Mesocosms were constructed with vinyl‐wrapped steel page‐wire garden fencing covered with fiberglass insect screen (Schmitz, 2008) and were deliberately placed to include equal abundances of the major functional plant groups. We arrayed the mesocosms (*n* = 50) throughout the old‐field in a randomized block design, with five nested treatments: (a) plants‐only, (b) plants with bold grasshoppers, (c) plants with shy grasshoppers, (d) plants with bold grasshoppers and a spider predator, and (e) plants with shy grasshoppers and a spider predator; for a total of ten replicates per treatment. Before stocking animals, we removed all other arthropods by hand and sealed the mesocosm. In early July, we collected individual *P. mira* sit‐and‐wait spiders from an adjacent field and stocked them into corresponding treatment mesocosms. By mid‐July, we stocked mesocosms with the respective bold and shy experimental populations at *n* = 5 individuals per mesocosm to simulate natural field densities (Schmitz, 2008). As sit‐and‐wait spiders, *P. mira* have low energetic demands and can go many months without eating, even to the extent that their chelicerae can be glued together without impacting their activity (Schmitz et al., 1997). In addition, despite our best efforts to remove arthropods, small organisms including mites and leaf hoppers were likely still present in the mesocosms, providing some alternative source of prey for the spider before the grasshopper populations were stocked. *P. mira* and the grasshoppers can both live in the mesocosm for the full duration of our experiment, with grasshoppers experiencing strong indirect effects of predation (Beckerman et al., 1997; Schmitz et al., 1997).

Grasshoppers cannot be individually identified because they molt twice between the third and fifth instar stages. Therefore, the level of replication for the field experiment was not the individual, but rather the population at the level of the mesocosm. Prior to stocking each mesocosm, we estimated (a) the population mean of personality and (b) the population variance in coefficient of relative plasticity (CRP). CRP gives a standardized index of intraspecific behavioral variation for an individual relative to its population (Reale & Dingemanse, 2010) and is calculated as:

$${\text{CRP}}_{i}=\frac{V_{i}}{V_{p}}$$

where *V*_i_ is the variance of the individual and *V*_p_ is the variance of the mesocosm population. The variance of CRP represents the range of behavioral plasticity within the population. Population mean of personality and population variance of CRP together render a more complete picture of the population‐level effects of intraspecific variation.

The field experiment ran from mid‐July to late September for a total of 70 days. Thereafter, we collected all surviving animals from the mesocosms and re‐assayed grasshoppers for population mean personality and variation of CRP using the same methods described above. We then calculated the change in mean personality trait and the change in CRP variance for each mesocosm. Post‐treatment repeatability estimates using the full 15min assays are similarly reported in the Appendix S1. Once animals were removed, we harvested all plant biomass and separated it into functional groups to measure trophic impact. Harvested vegetation was dried at 60°C for 48 hr and weighed. We standardized plant biomass relative to control treatments by subtracting control biomass from treatment biomass for each respective mesocosm block. The resultant difference value represented the degree of herbivory—negative values indicate less plant biomass and thus more herbivory, while positive values indicate more plant biomass and thus less herbivory.

We evaluated trophic impact, change in population personality trait mean, and change in population CRP variance using generalized linear mixed effects models (GLMM). Grasshopper survival and mesocosm treatments of personality, predation, and personality × predation were treated as fixed effects, with mesocosm block as the replicate random effect. Given the limited time span over which we conducted our personality assays, we also performed a rudimentary permutation test on change in population personality and change in population CRP to assess whether trends were driven by a statistical artifact of regression to the mean. Permutation tests for fixed effects in mixed effects models, to the best of our knowledge, do not yet exist. Therefore, we generated a random vector which permuted personality type and re‐ran our models to see if the results were the same as our experimental manipulation. All GLMMs were done in R (v3.4.3) with package lme4 (Bates, Mächler, Bolker, & Walker, 2015).

## 3 RESULTS

### 3.1 Intraspecific variation in personality trait

Individuals displayed repeatable activity within assays (*n* = 510; bootstrapped repeatability estimate = 0.599, *SE* = 0.021) and across contexts (*n* = 510; bootstrapped repeatability estimate = 0.607, *SE* = 0.024), at levels higher than an average repeatability of 0.37 estimated in a review of personality studies (Bell, Hankison, & Laskowski, 2009). The repeatability estimates for the three, 4‐min observations were not qualitatively different from the repeatability estimate for the full 15 min observation across contexts (bootstrapped repeatability estimate = 0.561, *SE* = 0.05, see Appendix S1). The frequency distribution of personality scores was strongly skewed right (Figure 3). We designated shy individuals to have personality trait values below the first quartile (8.13) and bold individuals to have personality trait values above the third quartile (36.84), meaning shy individuals had consistently low activity levels whereas bold individuals had consistently high activity levels across changes in environmental contexts. All others were considered to have intermediate shy–bold behavior (Figure 3).

Of the 30 individuals included in our long‐term laboratory experiment on lifetime consistency of personality assays, 29 survived for a minimum of two rounds of assessments, but only 10 survived for the full 70 days of the experiment. Mortality primarily occurred during molting events. Given that *M. femurrubrum* exhibit Type III survivorship (Beckerman et al., 1997; Schmitz & Suttle, 2001), this mortality rate is expected. For estimates of repeatability, we included all individuals that survived for a minimum of two rounds of personality assays. We found repeatability across assays and over time was low (three, 4‐min bootstrapped repeatability estimate = 0.128, *SE* = 0.037; 15‐min bootstrapped repeatability estimate = 0.051, *SE* = 0.05, see Appendix S1).

Grasshopper mass was not correlated with activity level (Pearson's *r* = −.08), but mass was slightly different between our classified personality types for the entire assayed population, with shy grasshoppers being 0.01 g larger than bold grasshoppers (Welch two sample *t* test; *n* = 510, *t* = −2.19, *df* = 249.86, *p* = .044; bold group mean = 0.048 g; shy group mean = 0.056). However, mass did not differ between the personality types for individuals included in field mesocosm study (Welch two sample *t*‐test; *n* = 200; *t* = −1.48, *df* = 122.1, *p* = .140, bold group mean = 0.049 g; shy group mean = 0.057 g); thus, we did not include mass as a population‐level variable in our mixed effects models on trophic impact.

Individuals used for respiration measurements and habitat domain assessment did not differ in mass (Welch two sample *t*‐test, *t* = −0.57, *df* = 28.64, *p* = .572; bold group mean = 0.11 g, shy group mean = 0.11 g). Note these experiments occurred after the third to fourth instar molt, so masses are expectedly larger than the masses reported above. Mass‐corrected respiration rates were not different between the personality types (repeated measures ANOVA, *n* = 32, *F*‐value = 2.79, *p* = .104). Respiration rates were higher in the non‐risk than in the risk situation (repeated measures ANOVA, *n* = 32, *F*‐value = 47.28, *p*‐value < .001), which is consistent with the expectation that acute exposure (<5 min) to a sit‐and‐wait predator in this prey species leads to short term suppression of respiration rates as the individual freezes to avoid immediate detection. Nonetheless, we did not find differences in physiology corresponding to personality type.

### 3.2 Personality and habitat domain

**FIGURE 4:**
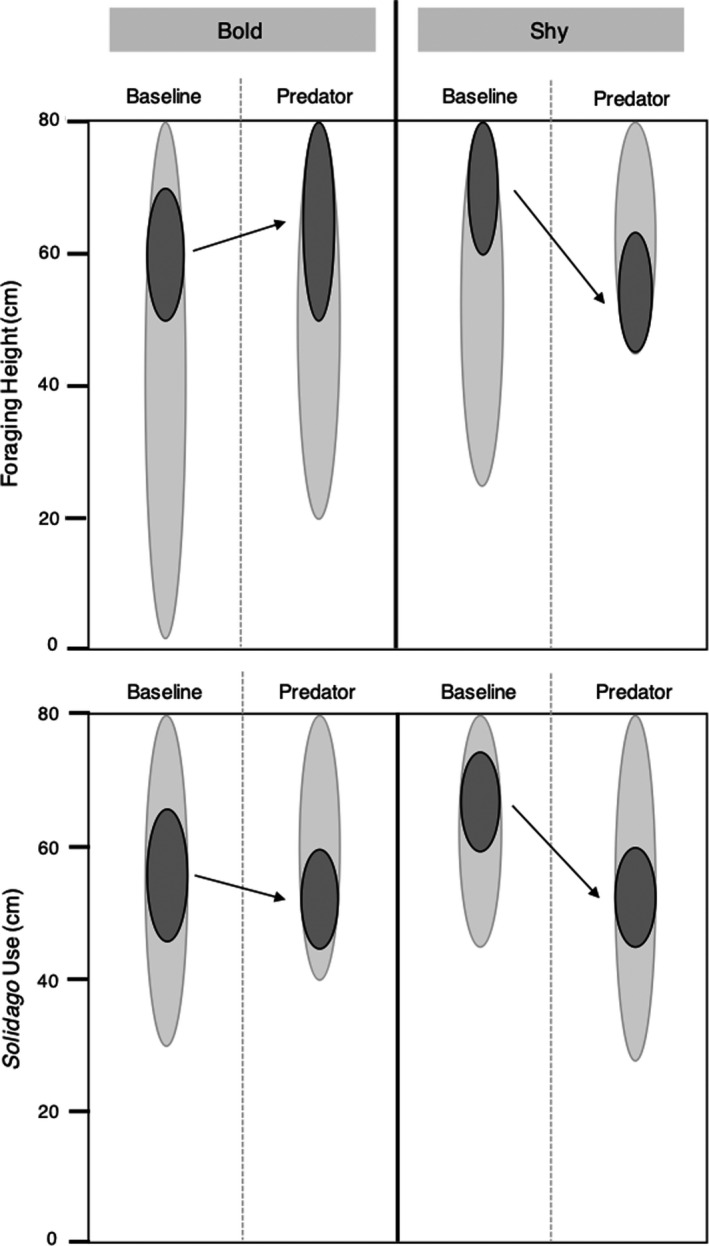
Spatial habitat domains of grasshoppers (*n* = 16 shy, *n* = 16 bold) with and without *Pisaurina mira* predation risk. The upper panel shows the vertical spatial extent of foraging movements in the vegetation canopy by individuals of the different personality types. The bottom panel shows the extent of movements specifically within *Solidago* vegetation. The ovals depict the 50% isopleths (dark grey) and 95% isopleths (light gray) of spatial utilization distribution. Arrows indicate net directional change in spatial location between predation risk contexts

Of the 40 total individuals that were placed into benchtop microcosms, three escaped and five died when they wedged themselves between the wooden posts and vinyl wrapping, resulting in habitat domain estimates for 32 individuals, 16 shy and 16 bold. Individuals used in the habitat domain experiment were the same as those used for respiration measurements; mass did not differ between the two personality types. In general, shy and bold individuals moved throughout the canopy to a similar extent (BA on 95% isopleth = 0.91, *χ^2^* = 31.25, *df* = 11, *p* = .001). While statistically different, the BA index indicates shy and bold individuals had near‐total overlap in the full extent of their movement. Core utilization was different between shy and bold individuals (BA on 50% isopleth = 0.47, *χ* ^2^ = 34.44, *df* = 2, *p* < .001). When a predator was present, bold individuals undertook greater forays to forage within the upper half of the canopy whereas shy individuals retreated lower down into the canopy (Figure 4 top panel, BA on 50% isopleth = 0.49, *χ* ^2^ = 23.17, *df* = 6, *p* < .001). The use of *Solidago* refuge habitat was also different between the personality types (BA on 50% isopleth = 0.11, *χ* ^2^ = 84.37, *df* = 5, *p* < .001); in absence of a predator, shy individuals occupied the upper 25% of the *Solidago* canopy. In the presence of a predator, shy and bold individuals both retreated lower into the *Solidago* canopy, although shy individuals moved considerably lower (Figure 4 bottom panel, BA on 50% isopleth = 0.36, *χ* ^2^ = 37.82, *df* = 3, *p* < .001).

### 3.3 Personality under predation

**FIGURE 5:**
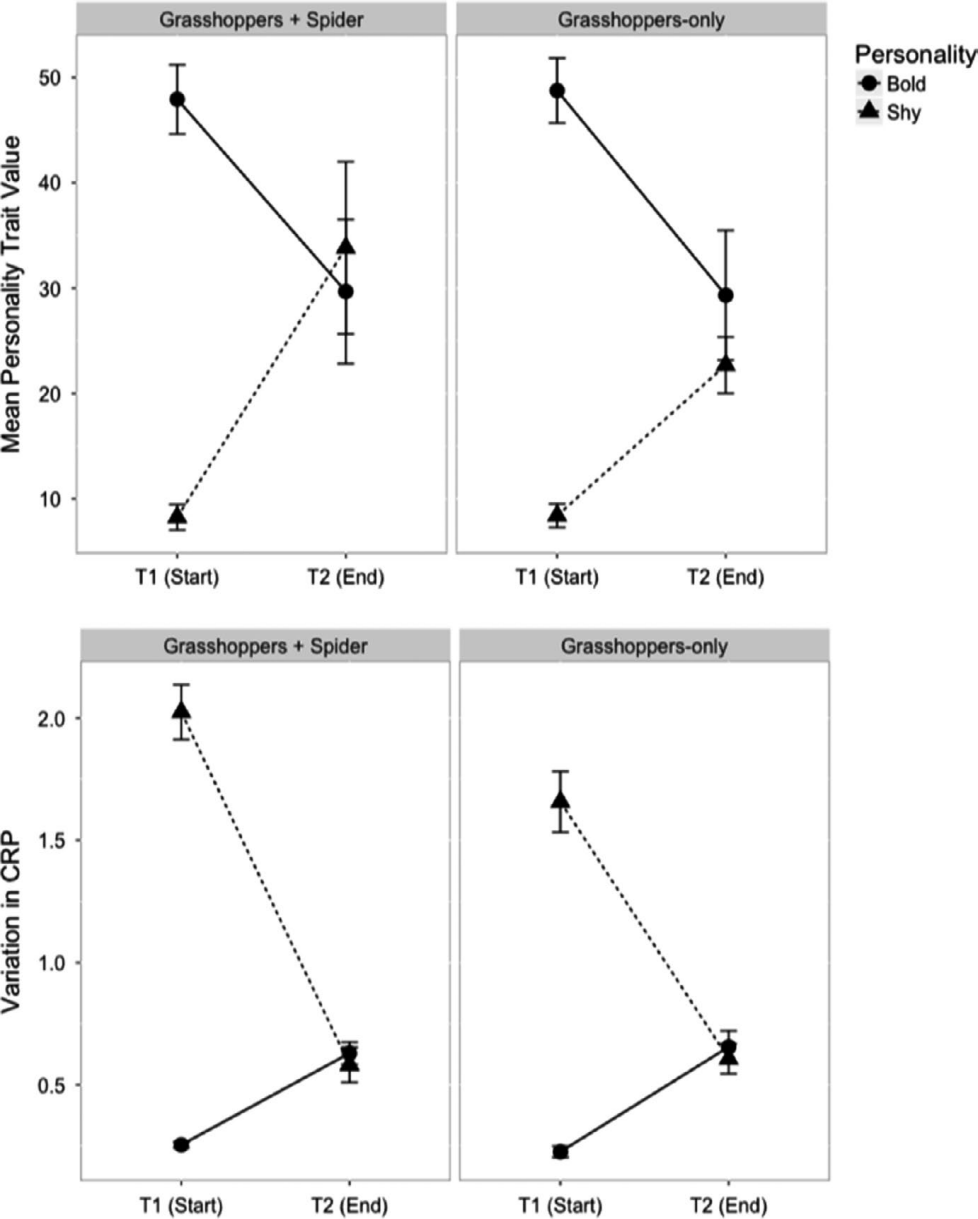
Personality trait values were not consistent for experimental populations (*n* = 50) between the start and end of the field study. For bold (circles) and shy (triangles) populations, the average trait value of the experimental population (top panel) at the start of the study was high and low, respectively. Values shown are the model estimates ± *SE*. At the end of the study, bold and shy populations converged toward trait values that were not significantly different from each other. This trend occurred irrespective of the predation treatment. Likewise, variation in individual plasticity for experimental populations (bottom panel) also changed between the start and end of the field study. Shy populations were generally more plastic than bold populations and converged toward similar levels of variation in plasticity

We did not find differences in grasshopper survival among treatments in the field experiment (ANOVA; *n* = 40, personality treatment: *F*‐value = 0.155, *p* = .695; predator treatment: *F*‐value = 3.522, *p* = .0686; personality × predation: *F*‐value = 0.669, *p* = .418). Mean survival was 2.42, with four mesocosms going extinct and three mesocosms having complete survival. Of the mesocosms that did not go extinct, change in mean personality for mesocosm populations was predicted by initial personality type (personality treatment: estimate = 43.85, *SE* = 10.48, *df* = 29, *p* < .001; predator treatment: estimate = −1.18, *SE* = 10.81, *df* = 29, *p* = .913, personality × predation: estimate = −10.10, *SE* = 15.57, *df* = 29, *p* = .521). Irrespective of predator treatment, shy populations shifted toward bolder trait values and bold populations shifted toward shyer trait values (Figure 5). Our permutation test suggested a low likelihood that regression to the mean drives this result (personality treatment: estimate = −16.98, *SE* = 19.131, *df* = 29, *p* = .382; predator treatment: estimate = −11.01, *SE* = 15.62, *df* = 29, *p* = .486; personality × predation: estimate = 18.22, *SE* = 25.308, *p* = .477), although given that individuals could not be tracked throughout the duration of this study, it perhaps cannot be ruled out completely. Change in the variation of plasticity (CRP) for mesocosm populations was also predicted by initial personality (estimate = −1.80, *SE* = 0.343, *df* = 25.76, *p* < .001). Bold populations became more plastic over the course of the field experiment while shy ones became less plastic. CRP results were also not sensitive to the same permutation test (personality treatment: estimate = 0.044, *SE* = 0.697, *df* = 29, *p* = .95; predator treatment: estimate = 0.22, *SE* = 0.569, *df* = 29, *p* = .702; personality × predation: estimate = 0.255, *SE* = 0.923, *p* = .784), similarly indicating that regression to the mean is not driving this result. Repeatability estimates from personality assays on surviving individuals was high (*n* = 86; within assays bootstrapped repeatability = 0.714, *SE* = 0.039; across assays = 0.715, *SE* = 0.390; 15‐min bootstrapped repeatability = 0.642; *SE* = 0.120; see Appendix S1).

### 3.4 Trophic impact

**FIGURE 6:**
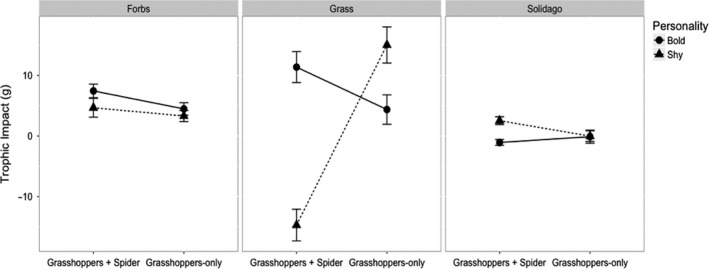
Each panel depicts the interaction between personality and predation on plant functional group biomass. The axis of trophic impact (g) was standardized to the control treatments within each block. Positive values indicate more plant biomass and less herbivory; negative values indicate less plant biomass and more herbivory. Values shown are the model estimates ± *SE*. Personality and predation influenced grass functional group biomass (middle), with shy grasshoppers (triangle) eating more grass in the presence of a spider predator and bold grasshoppers (circle) eating more grass in absence of a spider predator

Personality type and its interaction with predation predicted trophic impact on grass biomass (Figure 6, personality type on grass: estimate = −23.73, *SE* = 10.69, *df* = 31.05, *p* = .033; personality × predation on grass: estimate = 34.31, *SE* = 14.89, *df* = 31.09, *p* = .028). In absence of predators, bold individuals consumed more grass than shy individuals, but in the presence of predators, shy individuals consumed more grass than bold individuals (Figure 6). There was no effect of predation alone on grass biomass (estimate = −4.75, *SE* = 10.45, *df* = 31.02, *p* = .652) nor was there an effect of grasshopper survival on grass biomass (estimate = 4.49, *SE* = 2.72, *df* = 32.14, *p* = .108). There were no treatment effects on trophic impact for the other plant functional groups (see Appendix S1). CRP also did not have an impact on any plant functional group (see Appendix S1).

## 4 DISCUSSION

Functional trait approaches in ecology offer a way to mechanistically understand the link between the evolutionary ecology of organisms and their impacts on communities and ecosystems (Litchman & Klausmeier, 2008; McGill et al., 2006; Schmitz et al., 2015; Violle et al., 2007). These approaches have largely proceeded using the population mean value of the functional trait, but this may be insufficient given that trait variation among individuals within a population can lead to altogether different outcomes than would be expected based on the population mean alone (Figure 1; Benesh & Kalbe, 2016; Bolnick et al., 2011; Okuyama, 2008; Pettorelli et al., 2015; Post et al., 2008). Personality and its distribution within a population can have predictable consequences for the outcome of ecological interactions (Bell & Sih, 2007; Biro & Stamps, 2008; Dingemanse et al., 2010; Mcghee, Pintor, & Bell, 2013; Sih et al., 2004) and the effects of personality can cascade beyond focal predator–prey interactions (Belgrad & Griffen, 2016; Start & Gilbert, 2017). Hence, within‐population differences in personality ought to have a decided effect on trophic interactions. Our study examines how differences in prey personality manifest at the community level by studying its effects within a tri‐trophic food chain.

Our work revealed qualitative differences between shy and bold individuals in their spatial habitat domain under predation risk (Figure 4). Bold individuals foraged over a greater spatial extent than shy individuals and increased their core area of foraging under predation risk. Conversely, shy grasshoppers foraged at a lower height within the vegetation canopy, particularly under predation risk and retreated to a lower position in the *Solidago* canopy. *Solidago* is more structurally complex than grass and may act as a refuge under predation risk (Beckerman et al., 1997); by using *Solidago* and restricting their spatial extent of movement, shy individuals appeared to take less risks than bold individuals. In our study, assays for “shyness” were synonymous with consistently lower activity levels, but the ecological ramifications of that lower activity level were qualified using spatial habitat domain observations. Many personality studies identify consistent differences among individuals (e.g. Start & Gilbert, 2017), yet few have taken a mechanistic approach to ground personality types within ecologically relevant contexts (Carter et al., 2013; but see Kobler, Klefoth, Mehner, & Arlinghaus, 2009). Here, we identified personality types using assays over a very short time span, yet found important ecological differences in how those personality types move throughout the old‐field canopy. Canopy height in old‐fields is predictive of both plant nutrient concentrations (Barton & Schmitz, 2018) and predation risk, with predators of different functional hunting types selecting different vertical perch sites (Schmitz, 2005, 2007; Schmitz, Krivan, & Ovadia, 2004). Our spatial habitat domain observations yielded predictions for differences in trophic impact among the personality types.

We found that, in absence of predators, bold populations consumed more grass than shy populations, whereas in the presence of predators, shy populations consumed more grass than bold populations (Figure 6). From the perspective of prey physiological stress responses to predation risk (Beckerman, Wieski, & Baird, 2007; Clinchy, Sheriff, & Zanette, 2013; Hawlena & Schmitz, 2010b; Slos & Stoks, 2008; Thaler, McArt, & Kaplan, 2012; Zanette et al., 2011), this is an altogether unexpected result. Lower activity and use of *Solidago* habitat are typically considered fear responses by prey in this system (Hawlena & Schmitz, 2010a). *Solidago* is rich in soluble carbohydrates, providing readily assimilable energy to meet metabolic demands for increased escape performance, whereas grasses are protein‐rich, providing the building blocks for increased growth and development rates (Hawlena & Schmitz, 2010a). From our habitat domain observations, we might expect shy individuals to be more sensitive to predation risk and consume more carbohydrates, consistent with an animal stoichiometric perspective. However, our measure of shyness was based on activity level—lower energetic costs of activity would correspond with higher grass consumption for proteins that can be devoted to maximizing growth and reproduction.

We do not think differences in trophic impact were driven by differences in respiration rates or size between shy and bold individuals. From a behavioral standpoint alone, we may have inferred that shy individuals exhibited classic fear responses: lower activity level and higher risk‐aversion (Miller et al., 2014; Schmitz, 2007; Schmitz et al., 1997). However, there were no differences between shy and bold respiration rates, indicating personality types may not be equated with physiology, especially physiological response to perceived predation risk. We also found no differences in size between shy and bold individuals used in the mesocosm study, indicating size did not drive trophic impact.

In our mesocosm study, shy populations became bolder over the growing season, while bold populations became shyer (Figure 5). This may reflect regression to the mean due to the limited time span of our initial personality measurements (Barnett, van der Pols, & Dobson, 2005) and inability to track individuals within mesocosm populations, but our permutation test indicated that the change in mean personality over time was not entirely driven by regression to the mean. It is possible we may have seen a divergence in personality traits due to a pace‐of‐life syndrome (Stamps, 2007). Shy populations became bolder over the growing season (Figure 5) and concomitantly under predation, shy populations had a greater trophic impact on grass than bold populations (Figure 6). Grass consumption may be an adaptive dietary response by shy individuals under predation risk to increase their development rates. Rapid development rates correspond with increased body size, reaching both a predation size refuge from *P. mira* and sexual maturity by the end of the growing season (Abrams & Rowe, 1996; Ludwig & Rowe, 1990; Ovadia & Schmitz, 2002). Indeed, recent work has demonstrated that the direction and magnitude of the ecological effects of personality can change over development (Start, 2018), which correspond to changing environmental tradeoffs over an individual's lifetime. Additional work is certainly needed to test these hypotheses, but future research on personality and trophic impacts should consider interplay with other evolutionary ecological determinants of personality change such as life history imperatives driven by pace‐of‐life effects (Abrams & Rowe, 1996; Ludwig & Rowe, 1990; Moirón et al., 2020; Stamps, 2007).

We found no differences in trophic impact due to variance in relative plasticity (CRP). Similar to the population mean, CRP converged with more plastic mesocosms becoming less plastic and less plastic mesocosms becoming more plastic. Despite our null and inconclusive results, CRP is a potentially powerful metric to assess the relative contribution of the variance in trait means versus the variance in trait plasticity. Personality does not preclude plasticity (Dingemanse et al., 2010), but plasticity within personality has largely been explored through the lens of ontogeny (e.g. Edenbrow & Croft, 2011) and pace‐of‐life (e.g. Rádai, Kiss, & Barta, 2017). Trait plasticity can be more important than intraspecific variation in trait means for community‐level processes (Barbour et al., 2019), and personality studies can generate important insights when plasticity metrics such as CRP are included in their experimental design (Hall, Parson, Riebel, & Mulder, 2017).

We report high repeatability for our population personality assays, but low repeatability for personality across individual lifetimes. High repeatability for our personality assays is unsurprising because measurements for each individual all occurred within a 75 min timeframe and within the same experimental arena (Bell et al., 2009; Eccard & Herde, 2013; Garamszegi, Markó, & Herczeg, 2012; White, Schimpf, & Cassey, 2013). Low repeatability for the lifetime assays may be equally unsurprising due to laboratory habituation, individual variation in rates of habituation to assay stimuli, or simply variation across ontogeny (Bell & Peeke, 2012; Wuerz & Krüger, 2015). We also may have seen low repeatability due to cycles of fasting and feeding around the personality assays (Lichtenstein et al., 2016). Future work of this kind should be designed to assess differences in lifetime personality and individual trophic impact (i.e., diet) without being confounded by laboratory habituation. Nevertheless, our integrative study showed that individual differences captured in the personality assays were consistently manifested in habitat domains and in nature of variation of trophic impact.

We treated shy and bold as two different phenotypes, existing within the same consumer population, and explored the implications for trophic interactions (e.g., DeAngelis, 2013). Splitting the population into its extremes of shy and bold reveals the potential outcomes of trait variance when the range of intraspecific variation is ignored. The results of our mesocosm study are largely generalizable if and only if the population distribution of personality is bimodal, which was not the case. However, this design, focused on trait‐types, was necessary to test the general working hypothesis that distinct personality types do indeed have different effects. Such studies then become precursors to analyses that sample individuals from the entire trait distribution in ways that change trait variance rather than trait‐types and assess emergent community effects (Bolnick et al., 2011; Des Roches et al., 2018; Okuyama, 2008; Pettorelli et al., 2015; Post et al., 2008; Start & Gilbert, 2019; Toscano et al., 2020). Distributions of personality could vary locally, corresponding to plant community composition or variation in predator functional hunting types. Predation context can have divergent impacts on grasshopper behaviors (Figure 5), and predator functional hunting types vary across space and time (Miller et al., 2014; Schmitz et al., 2017). In response to changing trophic contexts, trait distributions could shift via plasticity or adaptation. As demonstrated in our study, differences in trait means can result in different dynamics under predator‐induced trophic cascades.

Our research shows that it is insufficient to characterize a population's trophic interactions across environmental contexts based on the mean trait value alone. That is, variation in trophic impact and, in our particular case convergence of personality trait values (Figure 1b), suggest that considering the state‐dependency of individuals within and among populations with different evolved trait values is needed to understand population responses to changing environmental contexts (Schmitz & Trussell, 2016). This reinforces previous arguments that understanding trait variance is important in understanding community‐level interactions (Bolnick et al., 2011; Des Roches et al., 2018; Pettorelli et al., 2015; Toscano et al., 2020). Our findings obviously beg for further experimental exploration of underlying mechanisms. Such examinations could offer deeper empirical insight into the nature of intraspecific behavioral variation within a population and how that variation scales to trophic interactions. As well, it would contribute to informing new theory that reconciles different behavioral types and their net trophic effects.

## CONFLICT OF INTERESTS

The authors declare no competing interests.

## AUTHOR CONTRIBUTION

**Nathalie R. Sommer:** Conceptualization (equal); Formal analysis (lead); Funding acquisition (lead); Investigation (lead); Methodology (equal); Resources (supporting); Writing‐original draft (lead); Writing‐review & editing (lead). **Oswald J. Schmitz:** Conceptualization (equal); Methodology (equal); Resources (lead); Writing‐review & editing (supporting).

## Supporting information

Appendix S1

## Data Availability

Data and code are available on Zenodo: https://doi.org/10.5281/zenodo.3955147.
