# Supplementary material for: Differences in prey personality mediate trophic cascades
Source: Ecol Evol. 2020 Aug 12;10(17):9538–51. doi: 10.1002/ece3.6648 (PMC7487229; doi:10.1002/ece3.6648)
Supplement: Supplementary file 1 — Appendix S1 [file ECE3-10-9538-s001.docx]

**Appendix**

*Unpartitioned repeatability estimates*

In our methods, we partitioned the 15 minute observation period for each assay into three, 4-min observations to obtain a measure of repeatability within assay contexts and across assay contexts. Here, we report the repeatability estimate for the full 15 minutes observation period across contexts in each instance of personality assessments: (1) the initial measurements to generate the personality distribution, (2) the re-assessment of post-treatment individuals, and (3) in the lifetime laboratory study*.

| Personality assessments | Bootstrapped repeatability | SE |
| --- | --- | --- |
| (1) Personality assessments for population distribution | 0.561 | 0.05 |
| (2) Re-assessment of post-treatment individuals | 0.642 | 0.12 |
| (3) Lifetime laboratory assessment | 0.051 | 0.05 |

*With 29 individuals surviving for a minimum of two rounds of measurements and 10 individuals surviving for seven rounds of measurements, there were 438 total observations. Repeatability was assessed with observation round and assay type as fixed effects.

Table of results for trophic impact by plant function type

| Plant functional group | Treatment | Estimate | SE | df | p-value |
| --- | --- | --- | --- | --- | --- |
| *Solidago* | Grasshopper survival | 0.48 | 0.94 | 33.37 | 0.613 |
|  | Predator treatment | 1.19 | 3.69 | 31.13 | 0.749 |
|  | Personality type | 3.45 | 3.77 | 31.21 | 0.367 |
|  | Personality × predator treatment | -3.04 | 5.25 | 31.30 | 0.567 |
| Forbs | Grasshopper survival | 0.29 | 1.16 | 31.99 | 0.799 |
|  | Predator treatment | -2.82 | 4.46 | 31.01 | 0.532 |
|  | Personality type | -3.50 | 4.56 | 31.04 | 0.449 |
|  | Personality × predator treatment | 3.04 | 6.36 | 31.08 | 0.636 |

Table of results for trophic impact by variation in CRP

| Plant functional group | Treatment | Estimate | SE | df | p-value |
| --- | --- | --- | --- | --- | --- |
| Grasses | Grasshopper survival | 3.83 | 3.00 | 32.23 | 0.210 |
|  | Predator treatment | 10.27 | 11.28 | 31.20 | 0.370 |
|  | Variation in CRP | -0.91 | 5.41 | 31.29 | 0.876 |
|  | Variation in CRP × predator treatment | 1.03 | 7.76 | 31.13 | 0.895 |
| *Solidago* | Grasshopper survival | 0.36 | 0.96 | 33.27 | 0.706 |
|  | Predator treatment | 2.12 | 3.64 | 31.44 | 0.565 |
|  | Variation in CRP | 1.59 | 1.75 | 31.59 | 0.369 |
|  | Variation in CRP × predator treatment | -2.32 | 2.51 | 31.31 | 0.362 |
| Forbs | Grasshopper survival | 0.33 | 1.14 | 31.98 | 0.770 |
|  | Predator treatment | -4.65 | 4.30 | 31.18 | 0.288 |
|  | Variation in CRP | -3.34 | 2.06 | 31.24 | 0.116 |
|  | Variation in CRP × predator treatment | 3.01 | 2.96 | 31.12 | 0.316 |
